# Agomelatine: A novel melatonergic antidepressant

**DOI:** 10.4103/0976-500X.72369

**Published:** 2010

**Authors:** S Manikandan

**Affiliations:** *Assistant Editor, JPP*

## INTRODUCTION

Depression is a devastating disorder, the treatment of which includes pharmacotherapy as well as psychotherapy. Mild depression can be treated with psychotherapy whereas moderate to severe depression requires pharmacotherapy. Despite the better understanding of the etiopathogenesis and availability of many antidepressants, treatment outcomes are not rewarding. Many patients either do not respond to treatment or have residual symptoms. Few others have intolerable adverse effects and become non-compliant or withdraw from therapy. Hence there is a need for newer antidepressants. Agomelatine is the latest in this category and belongs to an entirely new class. This article reviews the current status of this drug.

## MECHANISM OF ACTION

Agomelatine is a synthetic melatonin analog initially evaluated for its chronobiotic effect. Later when its effect on serotonergic receptors were discovered, it was investigated as an antidepressant.[1] It is an agonist at melatonergic MT_1_ and MT_2_ receptors and antagonist at serotonin 5HT_2C_ receptor.

Many hypotheses (monoamine hypothesis, neurotropic hypothesis, etc.) have been proposed to explain the pathophysiology of major depression. Phase-shift hypothesis is one among them and postulates that phase delay in internal circadian rhythm can lead to depression.[2] Stimulation of melatonergic (MT_1_ / MT_2_) receptors in suprachiasmatic nucleus of hypothalamus by agomelatine leads to the restoration of disturbed circadian rhythm. Hence it is referred to as ‘rhythm stabilizing antidepressant’.[3] By virtue of its selective binding to serotonin 5HT_2C_ receptors, agomelatine secondarily increases norepinephrine and dopamine levels.[2] This property also accounts for its antidepressant activity. It does not have any action on adrenergic, cholinergic and histamine receptors.

## PHARMACOKINETICS

Agomelatine is administered orally, undergoes extensive first pass metabolism and hence has a low bioavailability. It is extensively protein bound (95%) and its elimination half-life is 2.3 hours.[4] It is metabolized to a major extent (90%) by CYP1A2 and the rest by CYP2C9. It has no active metabolites and is excreted in urine.

## CLINICAL TRIALS

The clinical trials of agomelatine can be conveniently divided into short-term trials and long-term trials.

## SHORT-TERM TRIALS

The short-term trials are basically designed to evaluate the efficacy and safety except for one which is a dose-finding study. In all these trials, outcome is measured by Hamilton Depression Score. The dose-finding study conducted in 711 patients having major depression or bipolar II depression evaluated different doses (1, 5 and 25 mg) of agomelatine and found out that 25 mg is the most effective dose.[5]

Two trials each of 6-week-treatment duration evaluated the efficacy of agomelatine in a double blind placebo-controlled fashion. They had 212 and 238 patients with major depression, respectively. In both these trials treatment was started with 25 mg but increased to 50 mg if no improvement occurred after 2 weeks. Both the trials showed that agomelatine (two doses pooled) had a significant improvement when compared to placebo.

There are three unpublished short-term trials (6 weeks treatment period).[6] In each of these trials agomelatine was compared with placebo and an active comparator (fluoxetine or paroxetine). All these trials have shown that agomelatine is not significantly more effective than placebo.

## LONG-TERM TRIALS

A long-term trial involving 492 major depression patients followed them up to 24 weeks and concluded that agomelatine had a significantly lower cumulative relapse rate.[2] In a 12-week trial, agomelatine was compared with venlafaxine, the results of which revealed that the remission rate was comparable to venlafaxine.[4]

## ADVERSE EFFECTS

The commonly reported adverse effects in the clinical trials of agomelatine are headache, nausea and diarrhoea. It is found to increase the level of liver enzymes and so monitoring of enzyme level is warranted before starting therapy and thereafter every 6 weeks. It is also contraindicated in patients with hepatic impairment.[2] A meta-analysis of the treatment emergent sexual dysfunction with antidepressants has revealed that it has no significant difference with placebo.[7]

## DRUG INTERACTIONS

Relatively very less is known about the drug interactions as it is a new drug. Concomitant administration of CYP1A2 inhibitors like fluoxamine can lead to increased levels of agomelatine.

## CURRENT STATUS

Agomelatine is approved in Europe in 2009 for use in major depression. It is available there as 25 and 50 mg capsules. Clinical trials are in progress in USA and will be submitted for approval once these are completed. Currently it is not available in India.

## ADVANTAGES AND LIMITATIONS

Agomelatine possesses some distinct advantages. They are

- It restores biological rhythm and sleep without causing daytime sedation.
- It has minimal effect on sexual function.
- It is not addictive and there are no withdrawal symptoms.

Low oral bioavailability and propensity to elevate liver enzymes are its limitations.

## CONCLUSION

Agomelatine is a novel antidepressant with melatonergic (MT_1_/MT_2_) agonist and serotonin 5HT_2C_ antagonist property. It has provided a new approach to the treatment of depression. The published and unpublished literature show conflicting results. The trials have involved a few hundreds of patients only and the maximum follow-up period in the long-term trials is 24 weeks only. So the ‘real world’ effect of this drug is not known fully at this moment. It is available in Europe as 25 and 50 mg capsule for use in major depression. Trials designed for a head-to-head comparison with other SSRIs and second-generation antidepressants are needed. Finally to conclude, this drug has the potential to emerge as an alternate therapy for patients who are resistant to other antidepressants, but this needs to be tested.

## References

[1] Arendt J, Rajaratnam SM (2008). Melatonin and its agonists: An update. Br J Psychiatry.

[2] Howland RH (2009). Critical appraisal and update on the clinical utility of agomelatine, a melatonergic agonist, for the treatment of major depressive disease in adults. Neuropsychiatr Dis Treat.

[3] Fountoulakis KN (2010). Disruption of biological rhythms as a core problem and therapeutic target in mood disorders: The emerging concept of ‘rhythm regulators’. Ann Gen Psychiatry.

[4] Kennedy SH, Eisfeld BS (2007). Agomelatine and its therapeutic potential in the depressed patient. Neuropsychiatr Dis Treat.

[5] Eser D, Baghai TC, Moller H (2009). Agomelatine: The evidence for its place in the treatment of depression. Core Evid.

[6] Eurpean Medicines Agency. European Public Assessment Report – Valdoxon. http://www.ema.europa.eu/docs/en_GB/document_library/EPAR_-Summary_for_the_public/human/000915/WC500046224.pdf.

[7] Serretti A, Chiesa A (2009). Treatment-emergent sexual dysfunction related to antidepressants: A meta-analysis. J Clin Psychopharmacol.

