# ERRATUM

**Published:** 2011

**Journal of Pharmacology & Pharmacotherapeutics, 2010; 1: 122-3; Column 1, Paragraph 5, line 4.** **Title: Agomelatine: A novel melatonergic antidepressant** **The drug name ‘fluoxamine’** *Should read as* **‘fluvoxamine’**
The error is regretted ***- Chief Editor, JPP***

